# Processing and selection of surgically-retrieved sperm for ICSI: a review

**DOI:** 10.1186/s12610-017-0050-2

**Published:** 2017-03-21

**Authors:** Greta Verheyen, Biljana Popovic-Todorovic, Herman Tournaye

**Affiliations:** Centre for Reproductive Medicine, UZ Brussel, Laarbeeklaan 101, B-1090 Brussels, Belgium

**Keywords:** Testicular, Epididymal, Obstructive azoospermia, Non-obstructive azoospermia sperm motility, Sperm morphology, Sperm selection, ICSI, Testiculaire, Epididymaire, Azoospermie obstructive, Azoospermie non obstructive, Mobilité spermatozoïdaire, Morphologie spermatozoïdaire, Sélection des spermatozoïdes, ICSI

## Abstract

Although the technique of intracytoplasmic sperm injection (ICSI) has been a revolution in the alleviation of male infertility, the use of testicular sperm for ICSI was a formerly unseen breakthrough in the treatment of the azoospermic man with primary testicular failure. At the clinical level, different procedures of testicular sperm retrieval (conventional TESE, micro-TESE, FNA/TESA, MESA, PESA) are being performed, the choice is mainly based on the cause of azoospermia (obstructive versus non-obstructive) and the surgeon’s skills. At the level of the IVF laboratory, mechanical procedures to harvest the sperm from the tissue may be combined with enzymatic treatment in order to increase the sperm recovery rates. A number of techniques have been developed for viable sperm selection in males with only immotile testicular sperm available. However, large, well-designed studies on the benefit and safety of one over the other technique are lacking. Despite all the available methods and combinations of laboratory procedures which have a common goal to maximize sperm recovery from testicular samples, a large proportion of NOA patients fail to father a genetically own child. Advanced technology application may improve recovery rates by detection of the testicular foci with active spermatogenesis and/or identification of the rare individual sperm in the testicular suspensions. On the other hand, in vitro spermatogenesis or sperm production from embryonic stem cells or induced pluripotent stem cells might be future options. The present review summarizes the available strategies which aim to maximize sperm recovery from surgically retrieved samples.

## Résumé

Bien que la technique d’injection intra cytoplasmique d’un spermatozoïde (ICSI) ait constitué une révolution dans le soulagement de l’infertilité masculine, l’utilisation de spermatozoïdes testiculaires lors de l’ICSI fut une découverte capitale auparavant inaperçue du traitement de l’homme azoospermique par altération testiculaire primaire. Au plan clinique, différentes procédures de recueil des spermatozoïdes testiculaires sont réalisées (TESE conventionnelle, micro-TESE, FNA/TESA, MESA, PESA) dont le choix dépend principalement de la cause de l’azoospermie (obstructive versus non obstructive) et des compétences du chirurgien. Au plan du laboratoire de fécondation in vitro, des procédures mécaniques d’extraction des spermatozoïdes du tissu peuvent être combinées avec un traitement enzymatique dans l’objectif d’accroitre le taux de récupération de spermatozoïdes. Plusieurs techniques ont été développées pour sélectionner des spermatozoïdes vivants chez les hommes n’ayant que des spermatozoïdes testiculaires immobiles. Toutefois, il manque des études bien construites et à effectifs consistants portant sur le bénéfice et l’innocuité d’une technique par rapport aux autres. Malgré toutes les méthodes et combinaisons de procédures de laboratoire disponibles qui ont comme objectif commun d’accroitre au maximum la récupération de spermatozoïdes dans les échantillons testiculaires, un grand nombre de patients avec NOA échouent à obtenir un enfant qui soit génétiquement leur. L’utilisation d’une technologie de pointe devrait améliorer les taux de récupération par la détection de foyers testiculaires ayant une spermatogenèse active et/ou l’identification de rare spermatozoïde isolé dans les suspensions testiculaires. D’autre part, la spermatogenèse in vitro ou la production de spermatozoïdes à partir de cellules souches embryonnaires ou de cellules souches pluripotentes induites pourraient constituer de futures options. Le présent article de revue résume les stratégies disponibles qui visent à maximiser la récupération de spermatozoïdes dans des échantillons extraits chirurgicalement.

## Background

Although the introduction of intracytoplasmic sperm injection (ICSI) in 1992 was a major breakthrough in the treatment of male infertility [1], the successful use of epididymal and testicular sperm a few years later was a formerly unseen revolution in the treatment of the azoospermic man [2–4].

Azoospermia, defined as the absence of spermatozoa in the ejaculate after assessment of centrifuged semen on at least two occasions, is observed in 1% of the general population and in 10–15% of infertile men [5]. It can be clinically classified as either obstructive (OA, 40%) or non-obstructive azoospermia (NOA, 60%). Sperm retrieval is nearly 100% successful in OA patients, but the probability of finding sperm is around 50% in an unselected population of NOA patients [6]. While in OA a lot of sperm can be retrieved with epididymal aspirations (MESA, PESA), multiple testicular biopsies (TESE or micro-TESE) is the retrieval procedure of choice in NOA patients [7]. A Cochrane meta-analysis on surgical sperm retrieval techniques concluded that there is insufficient evidence to recommend any specific technique and that the least invasive technique should be used [8]. More recent reviews which focus on only NOA concluded that microsurgical TESE may be associated with a higher recovery rate. However, this conclusion should be interpreted witch caution due to the inclusion of low quality cohort studies [9, 10]. ART success rates after ICSI with testicular sperm may highly vary according to the cause of azoospermia and the selection of the patients.

Overall, non-obstructive azoospermia results in both lower fertilization and clinical pregnancy rates than obstructive azoospermia [11]. Additionally in NOA, the ART laboratory is challenged to efficiently process the tissue and to select those sperm with the highest potential to fertilize the oocyte and produce a competent embryo. The present review summarizes the evolution in the processing and selection of surgically retrieved sperm.

## Methods

An extensive computer search of PUBMED was performed using combinations of mainly the following search terms: “testicular sperm”, “epididymal sperm”, “non-obstructive azoospermia”, “obstructive azoospermia”, “immotile sperm,” “sperm selection”, “DNA fragmentation”, “mechanical”, “enzymatic”, “pentoxifylline”, “biregringence”. Reference lists of relevant articles and reviews were also analyzed for further articles. After review of titles and abstracts, references were limited to the original paper(s) describing the technique for the first time and/or to the more important papers related to the topic. A list of relevant articles that discussed laboratory techniques which are used to process and select sperm in the testicular samples as well as the future aspects was compiled and included in the review.

## Where do we come from?

### Epididymal sperm

In 1994 [3], Tournaye et al. reported successful fertilization and pregnancy rates with sperm obtained by microsurgical epididymal sperm aspiration (MESA) in patients with congenital bilateral absence of the vas deferens (CBAVD). MESA allows large sperm numbers with minimal contamination of red blood cells and non-germ cells to be retrieved. The aspirates are then simply emptied in a tube containing a buffered medium, and after mixing, a droplet is used to assess the quality of the spermatozoa (density, motility and estimation of morphology). If low numbers are obtained, the sample can be treated by double washing and centrifugation. In case of high numbers, density gradient centrifugation (90–45% or 80–40%) followed by double washing is preferred for selection of a motile fraction with better morphology. Ten μl droplets of the final sperm fraction are smeared on the bottom of a Petri dish and overlaid with oil. Motile sperm with good morphology are aspirated directly from this dish, immobilized and injected into the oocyte. In most cases, supernumerary sperm, left in the tube, can be frozen and successfully used in subsequent ICSI cycles [12]. Although high sperm numbers can be retrieved with MESA in almost all patients with obstructive azoospermia, ICSI technique is more successful than conventional IVF both in terms of fertilization and pregnancy rates [13].

Shortly after the introduction of MESA, percutaneous epididymal sperm aspiration (PESA) has been described as effective, simpler, and less traumatic alternative to MESA for patients with obstructive azoospermia [14, 15]. Nowadays, some units replaced the MESA procedure by PESA or testicular sperm retrieval. If performed by an experienced clinician, equally high sperm numbers may be obtained with PESA and MESA, and the processing of the obtained samples is similar.

### Testicular sperm

In the early nineties, the fertilizing capacity of human sperm directly retrieved from the testis has been discovered [2, 16]. Since then, the use of testicular sperm has become a routine procedure to circumvent infertility in patients with OA. A few years later this approach was also used to yield sperm in NOA patients [4, 17]. Although sperm retrieval and the outcome of ICSI are highly successful in OA patients, it remains a challenge in NOA patients, both at the clinical and the laboratory level. Notwithstanding several clinical characteristics (histopathology, FSH, Inhibin B) have been suggested, no single pre-operative parameter or combinations seem to accurately predict the ability to retrieve sperm surgically [6, 18–20]. Different surgical procedures are applied with different success rates according to the etiology of azoospermia, histological diagnosis and the surgical preference and expertise. While open testicular biopsy retrieval (TESE) or fine-needle aspiration (FNA/TESA) should guarantee sperm recovery in almost 100% of OA patients, TESE or micro-TESE [21] are the treatments of choice in NOA patients with the sperm recovery spans up to 40–50%, the micro-TESE potentially being 1.5 times more successful in retrieving sperm than TESE [10]. Considering that micro-TESE requires additional equipment and given low quality of evidence [9], multiple conventional TESE remains the most used procedure.

At the laboratory level, the embryologists are challenged to find sperm suitable for ICSI in the testicular tissue provided by the surgeon. In case of FNA/TESA performed in OA men, the aspirates are released in a tube or immediately in medium droplets under oil from which sperm are selected for ICSI. For TESE specimens, different procedures of processing have been described.

#### Mechanical processing of testicular tissue

Initially, different mechanical methods have been compared in order to maximize sperm recovery from testicular biopsies. Verheyen et al. [22] processed testicular tissue pieces by rough shredding, fine mincing, vortexing and crushing in a grinder with pestle. Fine mincing was the most effective method regarding the total number of motile sperm and normal morphology. Shredding and fine mincing of the tissue with rupture of the seminiferous tubules is so far the preferred and most widely used tissue processing procedure immediately after retrieval.

The excised testicular tissue is placed in a dish with buffered medium and shredded/minced with needles, microscope slides or scissors, by which sperm are released into the medium. The suspension is directly assessed under the inverted microscope at 200x or 400x magnification, or a drop is placed in a counting chamber and assessed with a phase-contrast microscope at 200x magnification. If sperm are easily identified, the suspension is transferred to a tube. After sedimentation of the remaining tissue pieces during 30–60 sec, the supernatant containing free sperm is further processed by centrifugation at 750xg for 5 min. Post-centrifugation, the supernatant is removed, the pellet is resuspended and 10 μl droplets are smeared on the bottom of a Petri dish and overlaid with oil. From this dish, the best-looking sperm (motility and morphology) are aspirated for ICSI. Zohdy et al. [23] described crushing of testicular biopsies quickly frozen in liquid nitrogen as an alternative method to improve sperm recovery. In order to avoid the presence of heterogeneous testicular cell types in the final fraction for ICSI, some groups successfully included a density gradient centrifugation step [24, 25]. In NOA patients, Kamal et al. [26] showed that in vitro microscopic dissection of the most dilated seminiferous tubules, if present, resulted in superior sperm recovery rates.

#### Erythrocyte-lysing buffer to facilitate sperm recovery

The excised testicular tissue is often highly contaminated with red blood cells which may hamper good visualization of spermatozoa in the suspension, especially if they are immotile. In order to solve this problem, the tissue suspension can be exposed to an erythrocyte-lysing buffer (ELB) [27, 28]. After tissue shredding, the suspension is centrifuged at 750xg for 5 min. The supernatant is aspirated and discarded, and the pellet is resuspended in 1 to 2 ml of ELB (155 mM NH_4_Cl, 10 mM KHCO_3_, and 2 mM ethylenediaminetetracetic acid EDTA; pH 7.2). The mixture is incubated at room temperature for 5 min. Next, the tube is filled up with buffered medium to dilute the ELB and is centrifuged at 750xg for 5 min. The supernatant is completely aspirated and discarded, and the pellet is resuspended in 0.5–1 ml buffered medium. From this fraction, a dish with 10 μl droplets under oil is prepared to perform ICSI. This technique enhances the efficiency of sperm collection, reduces the duration of sperm searching and improves the sperm selection process without negatively affect sperm fertilization potential after ICSI [28].

#### Enzymatic methods to improve sperm recovery

In azoospermic men in whom sperm production is very limited, especially in NOA patients, the combination of the limited tissue volume that can be removed, together with the low numbers of sperm present, renders mechanical extraction inadequate and the search for spermatozoa very complicated and time consuming. The use of enzymes to digest the testicular tissue that often hides the sperm is an efficient tool to improve sperm recovery. Both collagenase type IA and collagenase type IV have been successfully used to digest collagen as component of the basement membrane and extracellular matrix [28–30]. Incubation of the pelleted testicular suspension with collagenase IV rescued the ICSI treatment cycle in 7 out of 27 NOA men for whom no sperm was found after mechanical shredding [28]. Since then, the procedure is routinely applied in some centers after sperm detection failure following classical mechanical shredding. The enzyme solution consists of buffered medium with 1000 IU/ml collagenase IV, 25 μg/ml DNase and 1.6 mM CaCl_2_. A ready-to-use product is commercially available (GM501 Collagenase, Gynemed). After exposure of the tissue pellet at 37 °C during 1 h to 1–2 ml collagenase solution and meanwhile regularly shaking the tubes, the reaction is stopped by addition of 10 ml buffered medium. The digested tissue solution is gently centrifuged at 50xg for 5 min in order to remove residual non-digested pieces or debris. The supernatant cell suspension is washed twice with buffered medium and the resuspended pellet is used to prepare droplets under oil. In case of high numbers of red blood cells, ELB treatment is preceding the double washing step. Although not widely spread, several centers experienced the benefit of testicular tissue digestion [28, 31, 32].

This method can be considered adequate in maximizing sperm recovery and may increase the chance of selecting the highest quality sperm in NOA patients. Nevertheless, the procedure may still require several hours depending on the number of observed sperm and the number of oocytes to be injected. With regards to recovery rates reported after TESE, the use or non-use of enzymatic digestion may be one of the many confounding factors explaining the reported differences.

#### What about immature germ cells if no sperm are recovered?

The recovery of testicular sperm fails in at least 50% of NOA patients in unselected cohorts. Based on experiments in animal models, Edwards [33] suggested the use of round spermatids for ICSI if the more mature stages were not available. In 1995, the first successful births were reported by Tesarik [34] after round spermatid injection (ROSI) from the ejaculate. Since the mid-nineties, several IVF centers worldwide have used testicular round spermatids for ICSI despite the lack of preclinical evidence on safety and success. Problems of correct identification of the round spermatid in a wet preparation [35] and several basic concerns regarding their use for ICSI had been raised such as DNA immaturity, genomic imprinting problems, centrosome normality, presence of the oocyte-activating factor.

From a clinical point-of-view, maturation arrest at the round spermatid level is an extremely rare phenomenon, affecting only 0.9% of NOA men [36]. Birth of fewer than twenty children after ROSI have been reported worldwide, most of them derived from men who showed complete spermatogenesis, which casts doubt on the prevalence of a target population for ICSI with immature germ cells [35]. Data on ICSI with immature germ cells, and on in-vitro maturation of male germ cells and their safety concerns have been recently reviewed by Vloeberghs et al. [37].

## What progress did we make?

Nowadays, mechanical methods are still routine procedures used to recover sperm from testicular specimens, while the use of enzymatic methods is not widely spread, despite its proven effectiveness. Nevertheless, several challenges regarding the infertility treatment of men with NOA remain. At the clinical level, diagnostic parameters/tests which are able to fully predict testicular sperm retrieval are lacking. At the laboratory level, tools have been introduced to refine sperm selection in NOA patients.

Nagy et al. [38] demonstrated that the normal fertilization rate was significantly lower in cycles with only non-motile testicular sperm, and the difference was more pronounced in NOA cases than in OA cases. The causes and treatment options for complete immotility are reviewed by Ortega et al. [39]. Regarding testicular sperm, fertilization, pregnancy and implantation rates were negatively affected when only immotile sperm were available for ICSI in NOA patients [40, 41]. A number of methods for real-time selection of vital immotile sperm have been tested and introduced into clinical practice.

### Sperm culture

Some groups performed the surgical procedure one day prior to the oocyte retrieval in order to allow maturation of sperm with the goal to gain motility. They observed improvement of the motility during in-vitro culture, mainly in patients with OA [42, 43]. Balaban et al. showed the effectiveness of pre-incubating testicular sperm of 143 NOA patients in medium supplemented with recFSH [24]. Liu et al. [44] demonstrated that the results of in-vitro culture are unpredictable in cases with NOA.

In OA patients, the only effect observed was improvement of the quality of motility in samples that already showed motility shortly after tissue retrieval. Moreover, culture for more than 48 h seemed to cause sperm ageing, determined by increased DNA fragmentation and increased structural chromosomal abnormalities [45].

### Mechanical touch technique (MTT)

Since the introduction of ICSI, MTT has been used to distinguish vital from dead immotile sperm, if only immotile sperm were available for injection. The technique is based on the observation that immotile vital sperm have a flexible tail and immotile non-vital spermatozoa have a rigid tail upon lateral touching with the micro-injection pipette. If the tail is flexible and recovers its original position, the spermatozoon is considered viable. Tail rigidity and inability to recover the initial tail position is considered a sign of non-viability. In other words, if the head moves together with the tail, the spermatozoon is unsuitable for ICSI. Although this technique has been applied since the early years of ICSI, it has first been described by de Oliveira et al. in 2004 [46] as the sperm tail flexibility test. In both fresh and frozen TESE-ICSI cycles, they obtained similar fertilization rates with immotile and motile testicular sperm when applying MTT to select immotile sperm. Additionally, there were no differences in pregnancy and delivery rates between groups, indicating that the selection method was effective in identifying viable yet immotile testicular spermatozoa.

The strength MTT is that it does not change the integrity of the sperm, and no chemical substances are used, so that spermatozoa are ready to be injected. However, the technique is not 100% accurate and requires a certain level of expertise of the laboratory staff. Moreover, in cases with very few sperm, it may be very time-consuming to touch them one by one.

### Hypo-osmotic swelling test (HOST)

The hypo-osmotic swelling test was first developed by Jeyendran et al. [47] to evaluate the functional integrity of the sperm membrane. Viable sperm with a normal membrane function exhibit tail swelling or curling due to the influx of water when exposed to hypo-osmotic conditions. The use of this test to distinguish between viable and non-viable immotile ejaculated spermatozoa for ICSI was first proposed by Desmet et al. [48]. Tsai et al. [49] compared four hypo-osmotic solutions and found 150 mOsm NaCl to be the optimal HOS solution. A modified and simpler HOS test using culture medium and deionized grade water (1:1) was introduced by Verheyen et al. [50] who compared three different HOS solutions. After exposure for maximum 10 s to the mixture and identification of HOS-positive sperm, they can be aspirated and placed in an isotonic medium where sperm regain normal shape prior to injection. This modified HOS test has also been successfully applied to select immotile testicular sperm for ICSI. Fertilization rates and ongoing pregnancy rates increased from 28.2 and 2.9% in the control group (non-HOS) to 43.6% and 20.5% in the HOS group [51]. The HOS test has been described by the World Health Organization as alternative vitality test when sperm staining must be avoided [52]. However, false positive and false negative results reduce the test accuracy [53]. According to Hossain et al., the HOS method can not be considered a fully reliable alternative for vital staining because of spontaneously developed tail curling in fresh (5.9%) as well as in frozen (6.7%) sperm samples [54].

### Chemical motility enhancers

Pentoxifylline (PTF) inhibits phosphodiesterase activity and hereby increases levels of intracellular cyclic adenosine monophosphate (cAMP), a molecule which plays a role in sperm motility [55]. While Yovich et al. [56] introduced the use of PTF in clinical ART practice, Tasdemir and his group [57] were the first to show its effect on immotile testicular tissue samples as well. Because of safety concerns, the embryo toxicity of PTF was studied in a mouse model. This research revealed several adverse events which, however, could be avoided by washing sperm after their exposure to PTF [58]. It was found that addition of PTF to epididymal or testicular sperm not only initiated motility, but allowed fertilization, pregnancy and birth of healthy children after ICSI [59]. PTF was added to the sperm suspension in a 1:1 ratio, with a final PTF concentration of 3.6 mM. Addition of PTF in a lower final concentration (1.76 mM, 47 cycles) to immotile testicular sperm suspensions also induced motility and increased fertilization rates (66% vs 50.9%) and the number of available embryos (4.7 vs 2.7) after ICSI compared to control cycles (30 cycles), and pregnancy rate in the PTF group tended to be higher [60]. Also when frozen-thawed testicular samples were treated with PTF, significantly higher motility was observed [61]. The use of PTF for the selection of viable non-motile testicular sperm seemed more effective than use of the HOS test in a study by Mangoli et al. [62]. Irrespective of the heterogeneous data regarding clinical outcome, the use of PTF significantly reduces the time of sperm identification and selection.

Another member of the xanthine family, theophylline, has more recently been evaluated in a prospective trial on sibling oocytes and showed an immediate but short-term effect on sperm motility [63]. A ready-to-use product is commercially available (GM501 SpermMobil®, Gynemed). To a 10 μl droplet suspension smeared on the bottom of a dish, and overlaid with oil, 3 μl SpermMobil is added. The activating effect initiates within 10 min, and this effect lasts for maximum 1 h. Sperm that gained motility are aspirated and washed in sperm buffer prior to injection.

There have been concerns regarding the safety of these chemical compounds. In clinical settings where live births were reported following PTF or theophylline treatment, there is no evidence of anomalies in the offspring [64, 65], but, as always, larger follow up studies are needed.

### Laser-assisted immotile sperm selection (LAISS)

Aktan et al. [66] used the laser for the identification of viable but immotile spermatozoa. A single laser shot of 129 μJ for approximately 1.2 msec is directed to the tip of the flagellum which causes a coiling of the tail in vital immotile sperm. Conversely, if no tail displacement is observed, the sperm is dead. The number of viable sperm in a testicular sample identified by LAISS was comparable to that of the HOS test (22.0% vs. 21.5%) [66]. Fertilization rate improved, from 20.4% in the control group to 45.4% in the laser selection group. Accordingly, the take home baby rate increased from 5.9 to 19.0% [66]. Increased fertilization rate after laser-assisted selection of immotile testicular spermatozoa has been confirmed recently by Nordhoff et al. [67]. The use of the laser has the advantage that it does not require any chemical compound to induce motility, and thereby may be considered a safer approach. The main obstacle to its widespread application remains the high cost of the equipment needed [68].

### Birefringence –polarization microscopy (BPM)

This technique, first reported by Baccetti et al. [69], is based on the principle that viable sperm are naturally birefringent, based on their head structure, and dead sperm are not because of their different texture. Birefringence of the sperm head, as visualized with polarized light microscopy, reflects the health status of the cell and seems to correlate well with its viability [69]. In cases with severe male factor infertility, selection of immotile birefringence-positive sperm for ICSI resulted in higher clinical pregnancy rates (58% vs 9%) and implantation rates (42% vs 12%) than control-selected immotile sperm [70]. According to Ghosh et al., birefringence-based selection of immotile sperm was more effective in terms of pregnancy rate than HOS-selection (45% vs 11%) [71]. However, these conclusions are based on small studies, and larger studies are lacking. Moreover, the cost of the imaging equipment for BPM is high, while cheaper and easier methods for immotile sperm selection are available.

It can be concluded that, although several techniques have been developed to select viable sperm in cases with complete immotility, each has pros and cons. Large, properly controlled studies assessing the efficacy of the current techniques, are currently unavailable. Whatever technique is used, the success of ICSI with immotile sperm remains inferior to ICSI with motile sperm.

## Where are we now?

Twenty years after the introduction of the use of surgically retrieved sperm for ICSI, various retrieval techniques are still being performed, their choice depending on both the centre and the clinicians’ preference, and on the etiology of azoospermia.

While OA patients may benefit from PESA, FNA/TESA or eventually open TESE, multiple open excisional sampling, either by the conventional way or by using an operation microscope (micro-TESE), is the gold standard for NOA patients. In the latter population particularly, it is the embryologists’ challenge to optimize processing of the harvested tissue in order to find the rare sperm and select the vital and best-looking ones for micro-injection. It remains unclear what impact the laboratory phase has on sperm recovery rates reported in the literature, but this phase may be more important than the skills of the surgeon performing the retrieval. Different successful methods in order to select vital immotile sperm have been described, and the choice made by the IVF laboratory is guided by team expertise, grade of experience as well as equipment cost.

A number of processing methods are combined at the laboratory level in order to maximize sperm harvesting as well as the possibility to cryopreserve sperm for later use (not discussed in this paper). After initial shredding and mincing, enzymatic digestion of the tissue can be performed followed by a real-time sperm vitality testing whenever appropriate. The combination of enzymatic treatment with density gradient centrifugation and exposure to theophylline in order to maximize sperm retrieval has been applied by Wöber et al. [25]. A summary of procedures for processing and selection of surgically retrieved sperm is presented in Table 1.

**Table 1:** Overview of procedures for processing and selection of surgically retrieved sperm

	Clinical procedure	Laboratory procedure	Specific sperm selection procedure
Obstructive azoospermia	MESA/PESA	double washing or	
		density gradient centrifugation	
	TESA/FNA	immediate use	
	TESE	mechanical processing + washing	
Non-obstructive azoospermia	micro-TESE	mechanical processing +	
		immediate use	immotile sperm selection if needed
	TESE	mechanical processing + washing (or)	immotile sperm selection if needed
		mechanical processing + washing + ELB (or)	immotile sperm selection if needed
		mechanical processing + enzymatic processing	
		+ washing (+ ELB)	immotile sperm selection if needed:
			- MTT
			- HOST
			- LAISS
			- PTF/theophylline
			- LAISS
			- (BFM)

Although the birth of healthy children has been described for the different strategies of (immotile) sperm selection in NOA patients, these are mainly case reports or small cohort studies while larger longitudinal follow-up studies are lacking. Moreover, many aspects related to the individual sperm selected for ICSI remain unclear.

## Possible future aspects?

A number of issues regarding the use of surgically retrieved sperm, of NOA patients particularly, remain unresolved. Currently, longitudinal follow-up shows that more than two thirds of men with non-obstructive azoospermia do not realize their wish to become a biological father [72]. The only option for these couples is the use of donor sperm or adoption, which, for several reasons, may not be valid alternatives.

The question may be raised whether more sophisticated techniques which can more accurately determine focal spermatogenesis during surgery will improve this outlook. As for the lab phase, the use of automatic non-invasive sperm detection techniques on the harvested specimens may in the future increase sperm recovery rates together with a reduction in searching time. Whenever sperm are recovered, novel methods of sperm selection may improve the outcome after ICSI. Notwithstanding the progress made in the treatment of NOA patients, fertilization, embryo development, implantation rate and delivery rates are still far below those obtained with ejaculated sperm. Compared to OA with normal spermatogenesis, testicular sperm of NOA men is characterized by higher DNA fragmentation [73], increased aneuploidy rates [74], defects in spermiogenesis, and these features can currently not be identified at microscopic sperm selection for ICSI. Raman micro-spectroscopy has been suggested for in situ visualization of damaged DNA in living human sperm [75, 76], but this technique still requires further validation [77]. Increased mosaicism in the developing embryos suggests pre-implantation genetic screening (PGS) in this patient population. However, the PGS test nowadays implies biopsy of blastocyst cells, while many IVF centers perform replacement of cleavage-stage embryos (day 2/3) in ICSI cycles of NOA patients.

For NOA patients with unsuccessful sperm recovery, effective therapeutic options are currently not available, but extensive research in animal models might create future perspectives for male fertility restoration. Particularly for patients diagnosed with maturation arrest, in-vitro germ cell differentiation may offer a new therapeutic approach. Several groups study in-vitro spermatogenesis, either starting from testicular tissue culture [78], or from culture of isolated testicular germ cells or spermatogonial stem cells [79]. A variety of three-dimensional culture systems aiming to resemble the testicular micro-environment are currently tested. A promising novel option for spermatogenesis in vitro is the development of artificial scaffolds which mimic the spatial environment in the seminiferous tubules [80]. Although complete in-vitro meiosis of testicular germ cells and successful IVF after in-vitro spermatogenesis has been achieved in mice, its translation to the human remains a challenging issue for the time being. So far, culture conditions do not seem optimal for successful maintenance and proliferation of human spermatogonial stem cells. A recent report reviewed the current situation regarding human in-vitro spermatogenesis [81].

In-vitro production of sperm from embryonic stem cells or induced pluripotent stem cells has also been a focus of research for a number of decades. In mice, the generation of primordial germ cell-like cells, derived from embryonic stem cells and from induced pluripotent stem cells, with robust capacity for spermatogenesis has led to healthy offspring which developed normally and grew into fertile adults [82]. Progress and prospects are reviewed by Komeya and Ogawa [83]. However, this technique is far from being applied in the human for alleviating extreme male factor infertility. Besides the many unresolved biological aspects, safety concerns as well as ethical concerns require careful investigation.

## Conclusions

The use of ICSI with surgically retrieved sperm has moved frontiers in the fertility treatment of the azoospermic man. The sperm retrieval technique itself seems to have no impact on the success rates of ICSI. However, patients with NOA have lower sperm recovery rates and lower delivery rates than OA patients. The choice of the sperm recovery method from surgical samples and the method to select the individual sperm for ICSI depends on the embryologist and the laboratory equipment. Although several techniques are available to maximize sperm recovery and to select viable among immotile testicular sperm, only one in seven unselected NOA men has the chance to father a biological child. The struggle for improving the treatment outcome in NOA patients continues. Current research is focused on in vitro meiosis of testicular germ cells or spermatogonial stem cells, and on in vitro spermatogenesis, starting from induced pluripotent stem cells or somatic cells. However, many biological aspects remain unresolved, and safety and ethical concerns need careful investigation before its clinical application.

## Abbreviations

ART: Assisted reproductive technology
BPM: Birefringence-polarization microscopy
CBAVD: Congenital bilateral absence of the vas deferens
DNase: Desoxyribonuclease
EDTA: EthyleneDiamineTetrAcetic
ELB: Erythrocyte-lysing buffer
FNA: Fine-needle aspiration
FSH: Follicle stimulating hormone
HOST: Hypo-osmotic swelling test
ICSI: IntraCytoplasmic sperm injection
IVF: In Vitro fertilization
LAISS: Laser-assisted immotile sperm selection
MESA: Microsurgical epididymal sperm aspiration
MTT: Mechanical touch technique
NOA: Non-Obstructive azoospermia
OA: Obstructive azoospermia
PESA: Percutaneous epididymal sperm aspiration
PGS: Pre-implantation genetic screening
PTF: Pentoxifylline
recFSH: Recombinant FSH
ROSI: Round spermatid injection
TESA: Testicular sperm aspiration
TESE: Testicular sperm extraction
